# Funding map using paragraph embedding based on semantic diversity

**DOI:** 10.1007/s11192-018-2783-x

**Published:** 2018-05-28

**Authors:** Takahiro Kawamura, Katsutaro Watanabe, Naoya Matsumoto, Shusaku Egami, Mari Jibu

**Affiliations:** Japan Science and Technology Agency, Tokyo, Japan

**Keywords:** Paragraph embedding, Thesaurus, Information entropy, Map of science

## Abstract

Maps of science representing the structure of science can help us understand science and technology (S&T) development. Studies have thus developed techniques for analyzing research activities’ relationships; however, ongoing research projects and recently published papers have difficulty in applying inter-citation and co-citation analysis. Therefore, in order to characterize what is currently being attempted in the scientific landscape, this paper proposes a new content-based method of locating research projects in a multi-dimensional space using the recent word/paragraph embedding techniques. Specifically, for addressing an *unclustered* problem associated with the original paragraph vectors, we introduce paragraph vectors based on the information entropies of concepts in an S&T thesaurus. The experimental results show that the proposed method successfully formed a clustered map from 25,607 project descriptions of the 7th Framework Programme of EU from 2006 to 2016 and 34,192 project descriptions of the National Science Foundation from 2012 to 2016.

## Introduction

Price (1965) proposed studying science using scientific methods. Since then, studies have developed techniques for analyzing and measuring research activities’ relationships and constructed maps of science (Boyack et al. 2005), which is a major topic in scientometrics, to provide a bird’s eye view of the scientific landscape. Maps of science have been useful tools for understanding the structure of science, their spread, and interconnection of disciplines. By knowing such information, technology-driven enterprises can anticipate changes in the business environment. Research laboratories and universities that are organized according to the established disciplines can understand an organization’s environment. Moreover, such maps are important to policy analysts and funding agencies. Since research funding should be based on quantitative and qualitative scientific metrics, they usually perform several analyses on the map using statistical analysis with careful examination by human experts.

However, quantitative approaches to understanding research activities using inter-citation and co-citation analysis of published research papers focus on what authors told us about past accomplishments; thus, maps of science from literature characterize what was accomplished after a certain period of time for collecting a number of citations. Therefore, this paper focuses on what researchers currently want to work on their research projects. Research project descriptions do not have citations and references, and they cannot be analyzed using citation-based analysis including bibliographic coupling. We thus analyze them using a content-based method using the recent natural language processing (NLP) techniques, word/paragraph embedding. The embedding techniques represent words and paragraphs as real-valued vectors of several hundred dimensions. There were several content-based maps that are based on bag-of-words approaches in NLP. However, the paragraph embedding addresses the weaknesses of bag-of-words approaches by considering word orders; thus, it is considered to represent the semantics of the content more accurately.

We constructed a funding map using paragraph vectors as we described a preliminary version in Kawamura et al. (2017), In the map, nodes represent research projects that are linked by certain distances of the content similarity. Specifically, for addressing an *unclustered* problem associated with the original paragraph embedding technique, we introduce paragraph vectors based on information entropies of concepts in an science and technology (S&T) thesaurus. The main contribution of this paper is the construction of a content-based map characterizing what is being attempted in research projects based on the latest NLP techniques.

The remainder of this paper is organized as follows. “Related work” section discusses related work, and after the introduction of our experimental data set, “Measurement of project relationships” section  describes a baseline method and the proposed method for extracting relationships between research projects. Experiments and evaluations are described in “Experiments and evaluation” section, and conclusions and suggestions for future work are provided in “Conclusion and future work” section.

## Related work

Funding agencies and publishers generally have their own classification systems. In the 7th Framework Programme for European Research and Technological Development (FP7), most projects have three-digit subject index classification (SIC) codes^1^ that represent academic subjects, and some projects have multiple codes. Thus, interdisciplinary projects can be found by searching multi-labeled projects; however, even if two projects are assigned the same category, their similarities cannot be computed by the classification. Thus, it is also difficult to find the projects most similar to a new project. Moreover, funding agencies and publishers use different classification systems, and there is no comprehensive scheme for characterizing projects or articles; thus, they cannot be compared between different agencies or publishers. For example, comparing articles with the Association for Computing Machinery classification^2^ with the Springer Nature classification requires taxonomy exchanges.

The similarity between journals and articles can be calculated using the cosine and/or Jaccard similarity of inter-citation and co-citation (Boyack et al. 2005). Project descriptions, however, do not have citations and references. Therefore, citation analysis alone cannot be directly utilized for ongoing projects and recently published articles,^3^ although project descriptions will eventually include articles in their research results. Accordingly, Sci2 Team (2009), which provides common citation-based algorithms and effective visualizations to map articles and journals, also provides co-occurrence words and keywords extraction functions. The FP7 projects were visualized based on articles published by the project members in the Web of Science bibliographic database by examining co-authorship and co-citation patterns (Salah et al. 2013). Klavans and Boyack (2017a) combined citation-based and content-based approaches, in which 91,726 clusters (topics) created by clustering a direct citation network are located on a map based on content similarities of the topics, and also 314,000 project titles and descriptions retrieved from STAR METRIC^4^ are classified to the topics based on the content similarities.

In addition, several content-based methods are investigated in related literature. Previous studies have examined automatic topic classification using probabilistic latent semantic analysis (pLSA) (Steyvers and Griffiths 2007) and latent Dirichlet allocation (LDA) (Blei et al. 2003). One uses LDA to find the five most probable words for a topic, and each document is viewed as a mixture of topics (Griffiths and Steyvers 2004). This approach can classify documents across different agencies and publishers. However, the relationship between projects, such as that involving their similarity, cannot be computed directly. In this regard, the NIH Visual Browser (Talley et al. 2011; Herr II et al. 2009)^5^ computed the similarity between projects as the mixture of classification probability to each topic based on pLSA, using the average symmetric Kullback–Leibler divergence function (Kullback and Leibler 1951). However, this similarity is a combination of probabilities; that is, it is not derived from actual content semantics. Boyack et al. (2011) conducted a comprehensive study, in which they compared nine content-based similarity methods for clustering documents based on a large set of biomedical articles. Consequently, they concluded a BM25 approach (Jones et al. 2000) using titles and abstracts is superior to other approaches including tf-idf, LSA, and LDA, except for a PubMed’s own related article approach. However, their validation measure is about within-cluster textual coherence using the Jensen–Shannon divergence, and the similarity of pairwise articles is not measured. Wang et al. have recently proposed an approach using co-occurrence vectors with certain terms including topical terms extracted from titles and abstracts (Koopman et al. 2017; Wang and Koopman 2017). This can also be regarded as a refined version of the BM25 approach. Waltman et al. (2017) also conducted a study for comparing relatedness measures for clustering publications. They also concluded that BM25 yields more accurate clustering solutions than other text-based relatedness measures. Since Klavans and Boyack (2017a) also used BM25 for measuring content similarities, we compared our proposed approach with the BM25 approach in “Comparison with a BM25 apparoch on similarity of pairwise projects” section. In addition, there are studies combining citation-based approaches and content-based approaches (Ahlgren and Colliander 2009; Boyack et al. 2013). However, these hybrid approaches are out of the scope of this paper, since they also need citation information after all.

By contrast, a word/paragraph vector, which is a distributed representation of words and paragraphs, is attracting attention in NLP. Assuming that context determines the meaning of a word (Firth 1957), words appearing in similar contexts are considered to have a similar meaning. In the basic form, a word vector is represented as a matrix whose elements are the co-occurrence frequencies between a word *w* with a certain usage frequency in the corpus and words within a fixed window size *c* from *w*. A popular representation of word vectors is word2vec (Mikolov et al. 2013a, b). Word2vec creates word vectors using a two-layered neural network obtained by a skip-gram model with negative sampling. Specifically, word vectors are obtained by calculating the maximum likelihood of objective function *L* (Likelihood) in Eq. (1), where *N* is the number of words with a certain usage frequency in the corpus. Word2vec clusters words with similar meanings in a vector space.

1 $$\begin{aligned} L= & {} \frac{1}{N} \sum _{i=1}^{N} \sum _{-c \le j \le c, j \ne 0} \log Pr(w_{i+j} | w_{i}) \end{aligned}$$

Additionally, Le and Mikolov (2014) proposed a paragraph vector that learns fixed-length feature representations using a two-layered neural network from variable-length pieces of texts such as sentences, paragraphs, and documents. A paragraph vector is considered another word in a paragraph and is shared across all contexts generated from the same paragraph but not across paragraphs. The contexts are fixed-length and sampled from a sliding window over the paragraph. The paragraph vectors are computed by fixing the word vectors and training the new paragraph vector until convergence, as shown in Eq. (2).

2 $$\begin{aligned} L= & {} \sum _{i=1}^{N} \log Pr(w_{i} | w_{i-c},\ldots ,w_{i+c},p_{q}) \end{aligned}$$

where $p_q$ is a vector for a paragraph *q* that includes $w_i$. Whereas word vectors are shared across paragraphs, paragraph vectors are unique among paragraphs and represent the topics of the paragraphs. By considering word order, paragraph vectors addresses the weaknesses of bag-of-words models in LDA, pLSA, tf-idf, and BM25. Therefore, paragraph vectors are considered more accurate representations of the semantics of the content. We input resulting vectors into analysis of using machine learning and clustering techniques for finding similar projects in different academic subjects as well as the relationships between projects from different agencies.

## Measurement of project relationships

This section introduces our experimental data set and then describes a baseline method and the proposed method for extracting the relationships between research projects.

### Project description data sets

In this study, we analyzed all project descriptions of FP7, which can be freely obtained from European Commission’s primary portal of EU-funded research projects^6^ and part of project descriptions of the National Science Foundation (NSF), which can also be freely obtained at NSF website.^7^

Precisely, the FP7 data set consists of the titles and descriptions of 25,607 projects from 2006 to 2016, including 305,819 sentences in total. The NSF data set consists of the titles and descriptions of 34,192 projects from 2012 to 2016, including 730,563 sentences in total. Since the number of NSF projects is much larger than that of the FP7 projects, we limited the research areas to Computer & Information Science & Engineering, Mathematical & Physical Sciences, and Engineering for 5 years. All words in the sentences were tokenized and lemmatized before creating the vector space. This paper used the project descriptions, but our content-based approach can handle project descriptions and article abstracts in the same way.

### Baseline method using paragraph vector

Before introducing the proposed method, we present a problem in applying the paragraph vectors for research project descriptions. We implemented the paragraph embedding technique using the Deep Learning Library for Java.^8^ Then, we constructed paragraph vectors for FP7 and NSF projects, respectively. Although we need a more systematic way, but this time the hyperparameters were set empirically as follows. To fix the number of dimentions, we conducted principal component analysis on several hundred paragraph vectors. As a result, we found that the cumulative proportion reaches more than 90% at around 400 dimensions in our data sets. Thus, 500-dimensional vectors including a margin were established for words that appeared more than five times; the window size *c* was 10. the learning rate and minimum learning rate were 0.025 and 0.0001, respectively, with an adaptive gradient algorithm. The learning model is a distributed memory model with hierarchical softmax.

**Fig. 1:**
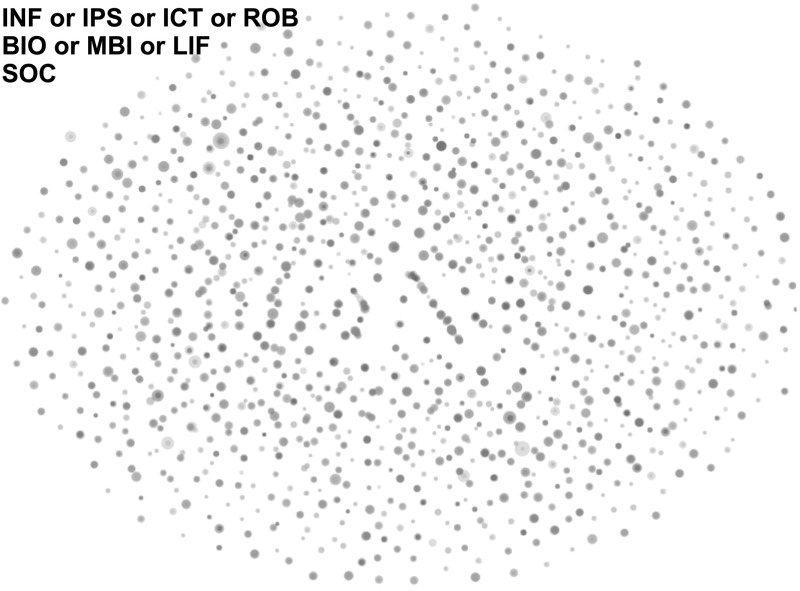
FP7 projects that have the cosine similarity of > 0.35 to other projects (*BIO* Biotechnology, *MBI* Medical Biotechnology, *LIF* Life Science, *SOC* Social Aspects, *INF* Information and Media, *IPS* Information Processing and Information Systems, *ICT* Information and Communication Technology Applications, *ROB* Robotics). (Color figure online)

In Fig. 1, each node represents a FP7 project, and the distance between projects corresponds to the cosine similarity of the paragraph vectors. However, we found that projects are scattered and not clustered by any subject or discipline in the vector space. Most projects are slightly similar to a low number of projects. Thus, it was difficult to grasp trends and compare an ordinary classification system such as SIC codes. The map of the NSF projects also had the same tendency.^9^ Closely observing the vector space reveals some of the reasons for this *unclustered* problem: each word with nearly the same meaning has slightly different word vectors, and shared but unimportant words are considered the commonality of paragraphs. In fact, Le et al. reported a classification accuracy with multiple categories of less than 50% (Le and Mikolov 2014). Therefore, for addressing this problem, we propose paragraph embedding based on the semantic diversity of concepts defined in a thesaurus, aiming to make paragraph vectors meaningful groups from the scientific and technological point of view. This approach can also be considered as an introduction of explicit semantics in a thesaurus to implicit semantics of a vector representation.

### Entropy-based paragraph vector

The fact that synonyms tend to gather in a word vector space indicates that the semantics of a word spatially spread to a certain distance. This observation is also suggested in the related literature (Vilnis and McCallum 2015). Therefore, to unify word vectors of almost the same meanings, excluding trivial common words, we generate clusters of word vectors based on the semantic diversity of each concept in an S&T thesaurus.

**Fig. 2:**
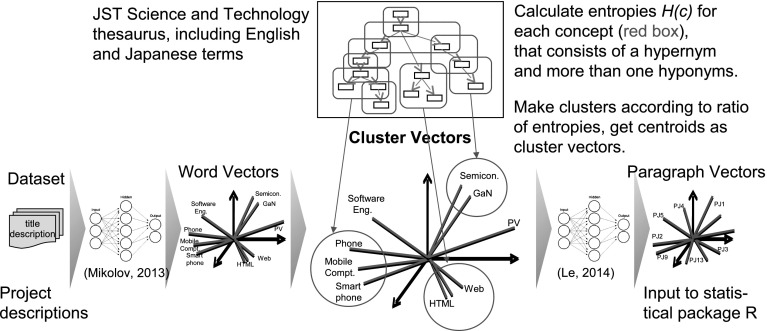
Construction of paragraph vectors based on cluster vectors

We first extract 19,685 concepts with one or more hyponyms from the JST thesaurus (Kimura et al. 2015). The JST thesaurus primarily consists of keywords that have been frequently indexed in 36 million articles accumulated by the JST since 1975. Currently, this thesaurus is updated every year, and includes 276,179 terms with English and Japanese notations in 14 categories from bioscience to computer science and civil engineering. Based on the W3C Simple Knowledge Organization System (skos), the JST thesaurus also exists in Linked Data form with semantic relationships skos:broader, skos:narrower, and skos:related. A broader or narrower relationship essentially represents an *is-a* subsumption relationship but sometimes denotes a *part-of* relationship in geography and body organ terminology. The JST thesaurus is publicly accessible from Web APIs on the J-GLOBAL website,^10^ along with the visualization tool Thesaurus Map.^11^ We then calculate the information entropy (Shannon 1948) of each concept in the JST thesaurus from the FP7 and NSF data sets, respectively. *Shannon’s* information entropy in information theory is an estimate of event informativeness. We used this entropy to measure the semantic diversity of a concept (Santus et al. 2014). After creating clusters according to the magnitude of entropy, we unify all word vectors in the same cluster to a cluster vector and constructed paragraph vectors based on the cluster vectors. The overall flow is shown in Fig. 2.

3 $$\begin{aligned} H(C) = - \sum _{i=0}^{n} \left( \sum _{j=0}^{m} Pr(S_{ij} | C) \cdot \log _2 \sum _{j=0}^{m} Pr(S_{ij} | C) ) \right) \end{aligned}$$

**Fig. 3:**
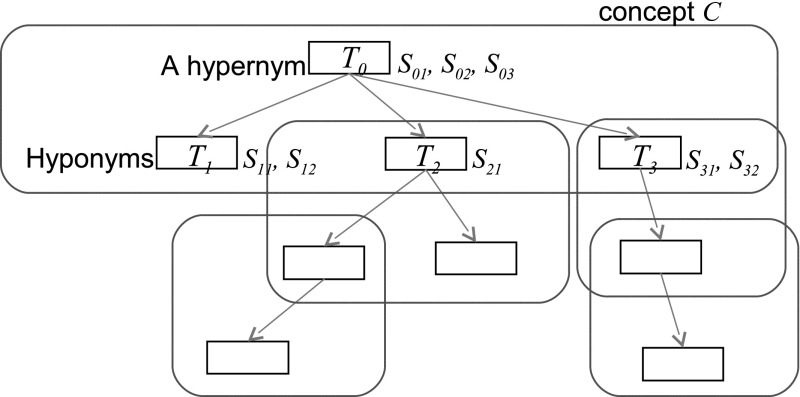
Concepts in a thesaurus. (Color figure online)

Specifically, the “word” is a word in the data sets, the “term” is a term in a thesaurus, and terms are classified into hypernyms, hyponyms, and their synonyms. The “concept” is defined as a combination of a hypernym and one or more hyponyms one level below the hypernym, indicated as a red box in Fig. 3. Given that a thesaurus consists of terms $T_i$, we calculated the entropy of a concept *C* by considering the appearance frequencies of a hypernym $T_0$ and its hyponyms $T_1...T_n$ as an event probability. The JST thesaurus has a hierarchical structure, but hyponyms only one level below the hypernym were included in the event probability to prevent the entropies of upper-level hypernyms being too high. The frequencies of synonyms $S_{i0} \ldots S_{im}$ of term $T_i$ were summarized to a corresponding concept (synonyms $S_{ij}$ include descriptors of terms $T_i$ themselves). In Eq. (3), $Pr(S_{ij}|C)$ is the probability of a synonym $S_{ij}$ given a concept *C* and terms $T_i$. For each concept in the thesaurus, we calculated the entropy *H*(*C*) in the data sets. As the probabilities of events become equal, *H*(*C*) increases. If only particular events occur, *H*(*C*) is reduced because of low informativeness. Thus, the proposed entropy of a concept increases when a hypernym and hyponyms that construct a concept separately appear with a certain frequency in the data set. Therefore, the magnitude of entropy indicates the semantic diversity of a concept. For example, the high-entropy concepts in the FP7 data set include relatively abstract *property, object, protein, material, polymer, method, equipment*, etc. The low-entropy concepts include more specific *soil stabilization, computer assisted diagnosis, hydropolyoxide, pulmonary mycosis*, etc. Then, assuming that the magnitude of entropy and the spatial size of a concept in a word vector space are proportional to a certain extent, we split the word vector space into several clusters. In fact, our preliminary experiment indicated that the entropy of a concept has high correlation *R* = 0.602 with the maximum Euclidean distance of hyponyms in the concept in a vector space, at least while the entropy is high. Specifically, we refined clusters by repeatedly subdividing them until the defined criterion was satisfied. In our method, we set the determination condition as shown in Eq. (4).

4 $$\begin{aligned} Cl(w_k) = {\left\{ \begin{array}{ll} Cl(w_{i}) &{} \left(\frac{H(C(w_i))}{H(C(w_j))} > \frac{\Vert w_k-w_i\Vert }{\Vert w_k-w_j\Vert } \right) \\ Cl(w_{j}) &{} ({\mathrm{otherwise}}) \end{array}\right. } \end{aligned}$$

This condition represents that the word vectors $w_0 \ldots w_N$ are subdivided into two clusters proportionally to the ratio of the highest two concept entropies $H(C(w_i))$ and $H(C(w_j))$, which are selected from all entropies of concepts in a cluster (an initial cluster is the whole vector space). $C(w_i)$ and $C(w_j)$ mean concepts *C* to which words $w_i$ and $w_j$ belong, respectively. The words $w_i$ and $w_j$ are words, whose lemmatized forms are indentical to terms or synonyms in the thesaurus. The entropies of words that are not included in the thesaurus are not calculated in Eq. (3). However, even if words in the description do not match to any term or synonyms in the thesaurus, the paragraph vector for the description is created since every word will be an element of a cluster of any concept in the thesaurus. *Cl*(*w*) means a cluster to which a vector of a word *w* should be classified.

The vector space is subdivided until the entropy becomes lower than 0.25 (the top 1.5% of entropies) or the number of elements in a cluster is lower than 10. These parameters were also determined empirically through the experiments. After generating, e.g., 1,260 clusters from 66,830 word vectors in the FP7 data set, we considered the centroid of all vectors in a cluster as a cluster vector. Then, we constructed paragraph vectors using the cluster vectors rather than word vectors, as shown in Eq. (5) that is an extension of Eq. (2): After all, each cluster vector represents a concept that has the highest entropy in all concepts included in the cluster.

5 $$\begin{aligned} L = \sum _{t=1}^{T} \log Pr(Cl(w_{t}) | Cl(w_{t-c}),\ldots ,Cl(w_{t+c}), p_{q}) \end{aligned}$$

## Experiments and evaluation

**Fig. 4:**
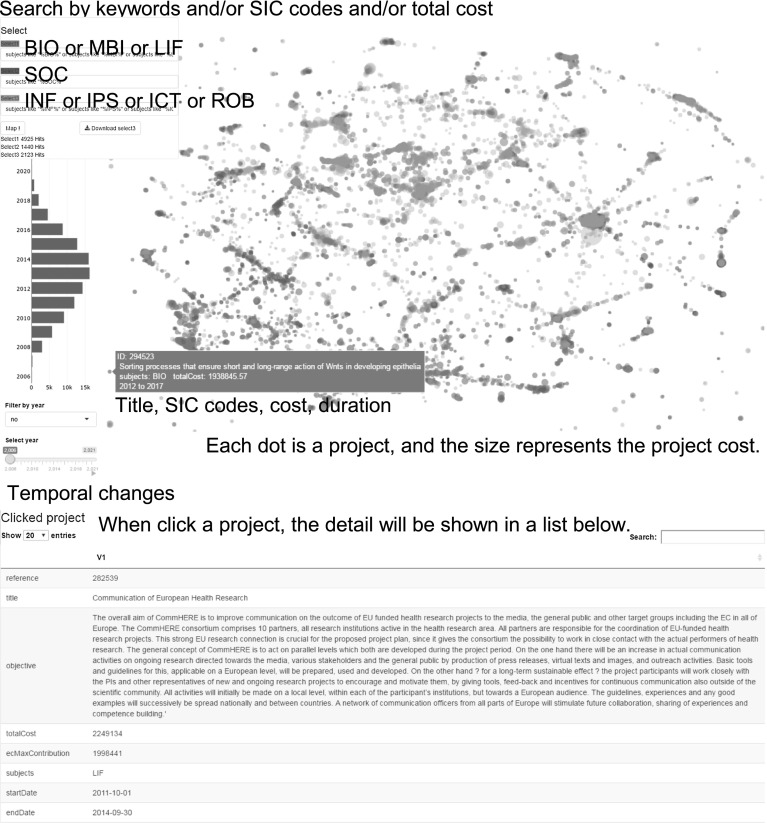
Funding map of FP7 projects that have a cosine similarity of > 0.35 to other projects. (Color figure online)

The funding map for FP7 is illustrated in Fig. 4 and publicly accessible on our website.^12^ The map for NSF is not shown in this paper, but also publicly accessible on our website.^13^ The OpenOrd^14^ that produces edge-weighted graphs, was used to locate the projects according to thier distances. The OpenOrd produces a layout, wherein similar nodes are placed as close to one another *as possible*, and shows the local clustering of related nodes and the global structure of related clusters. We computed 328 million cosine similarities for all pairs of the 25,607 FP7 projects and 11.7 billion cosine similarities for all pairs of the 34,192 NSF projects; however, we kept only those that were above a given threshold (0.35 in the figure) as edges, since the number of edges exponentially increases after the threshold.

In the map, a keyword search is available for project titles, descriptions, and SIC codes. Each node represents a project, and the size of the node corresponds to the project cost. When a mouse is pointed to a node, the project title, cost, and SIC code appear. When a node is clicked, the details including the project description appear below the map. A time bar on the left side represents the start year of the projects, and the map can move along the time bar. Since each node is coupled with a publication month/year, we can zoom in the specified time window to detect emerging trends.

### Comparison with the baseline on the *unclustered* problem

Figures 1 and 4 illustrate the FP7 maps obtained by the baseline and the proposed methods with the same hyperparameters, respectively.^15^ In Fig. 1, project nodes were scattered, and thus when we perform clustering on the map as in “Comparison with SIC codes” section, the variances, such as within-cluster sum of squares in k-means clustering get worse, and the cluster quality becomes low. For this *unclustered* problem, we can confirm that Fig. 4 of the proposed method formed several groups based on the content similarity.

**Fig. 5:**
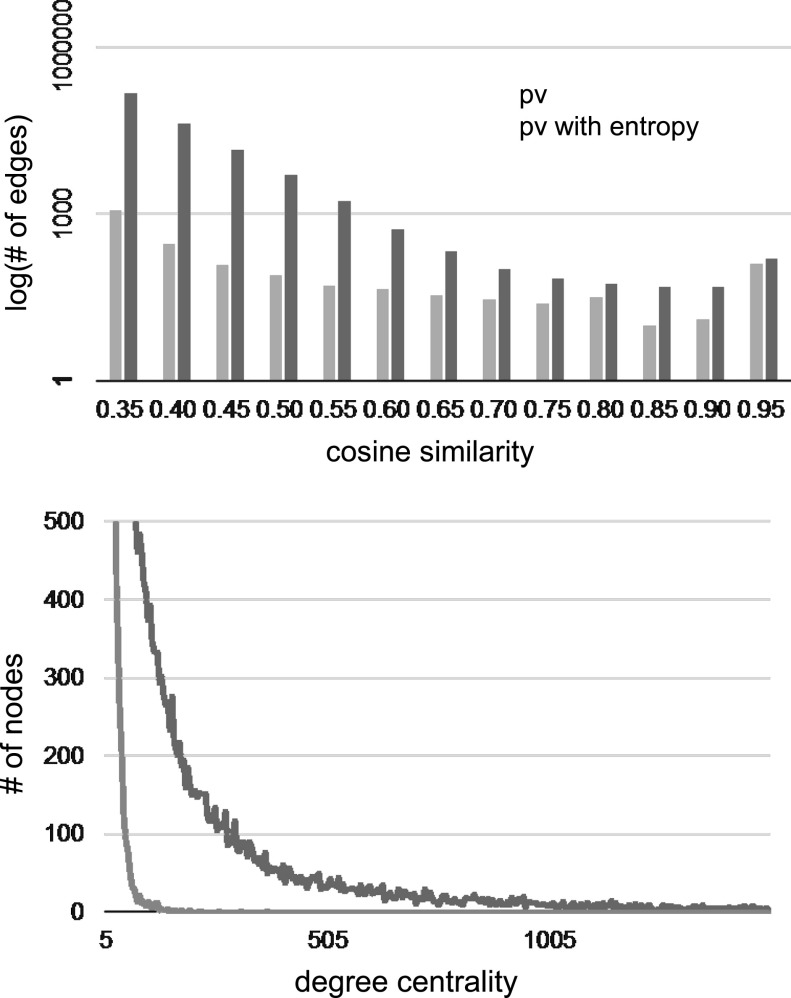
Edges with higher similarities and nodes with higher degree centrality in the original paragraph vectors and entropy-clustered paragraph vectors from the FP7 data set

**Fig. 6:**
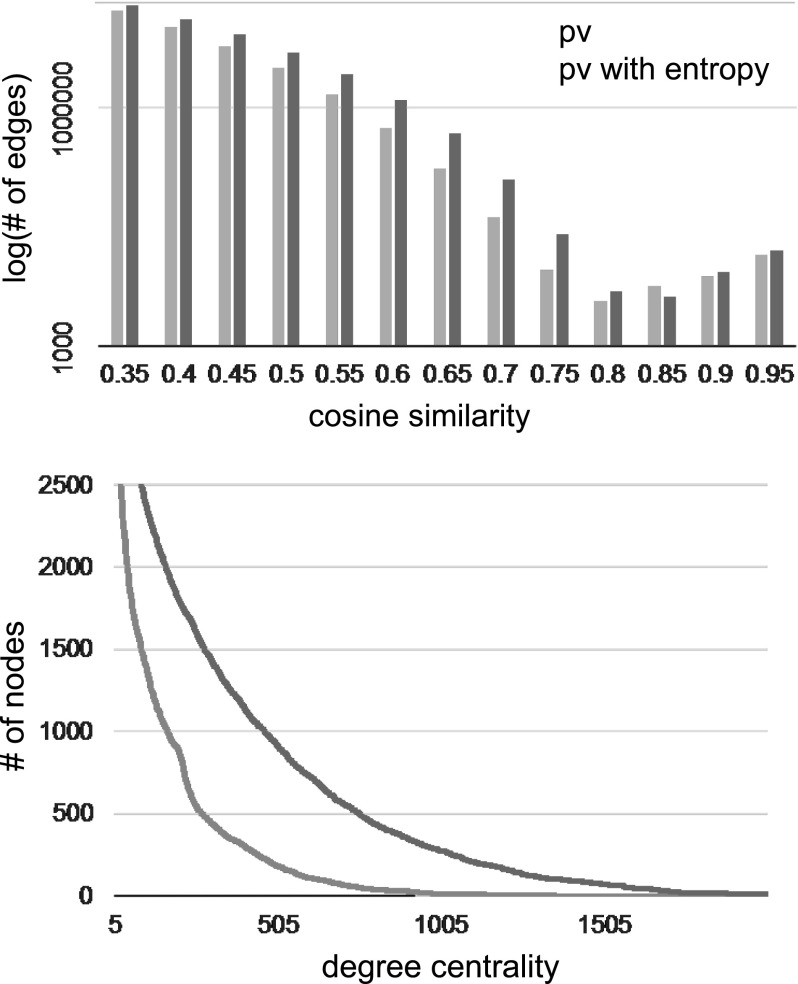
Edges with higher similarities and nodes with higher degree centrality in the original paragraph vectors and entropy-clustered paragraph vectors from the NSF data set

For more quantitative comparison, Fig. 5 for the FP7 data set and Fig. 6 for the NSF data set present the relationships between the cosine similarities and the number of edges, and the relationship between the degree centrality and the number of nodes (i.e., projects) in the case of the cosine similarities of > 0.35. In the graphs, we confirmed that both edges with a higher cosine similarity and nodes with higher degrees increase in the proposed method. The reason for this result is because through the use of high-entropy concepts as common elements between paragraph vectors, the paragraph vectors were able to comprise meaningful groups in the S&T contexts. Simultaneously, unknown synonyms and closely related words in the same cluster, which are not included in the thesaurus were unified to a cluster vector. Taking the centroid vector as a representative vector in a cluster involves separating each cluster vector as much as possible to form a clear difference in the vector space. Comparing the FP7 map and the NSF maps, we found that the NSF map is composed of much more edges with higher similarities and nodes with higher degree centrality than the FP7 map, since the NSF data set is larger than the FP7 and also limited to relatively close research areas, that is, Computer & Information Science & Engineering, Mathematical & Physical Sciences, and Engineering.

### Comparison with a BM25 apparoch on similarity of pairwise projects

The accuracy of the project similarity is important, since the map is used for real-world problems of research planning and evaluation. However, the evaluation encounters difficulty since, to the best of our knowledge, there is no gold standard for evaluating the similarity among S&T documents. Therefore, we first evaluated the accuracy of the similarities based on a sampling method. We randomly selected 100 project pairs that have a cosine similarity of $\ge$ 0.5 from the FP7 and NSF maps.^16^ The distribution of disciplines in the sample pairs was randomized, thus we assumed that it was almost the same as the distributions of the data sets. Each pair has two project titles and descriptions, and then we divided cosine values of the pairs into three levels: *weak* (0.5 $\le$ cos. < 0.67), *middle* (0.67 $\le$ cos. < 0.84), and *strong* (0.84 $\le$ cos.). Then, three members of our organization, a funding agency in Japan, evaluated the similarity of each pair. The members were provided the prior explanations for the intended use of the map and some examples of evaluation. The members received the same pairs, and their backgrounds are bioscience, psychology, and computer science. Then, they classified the 100 pairs to *weak, middle, strong*, and *not-related*.

We here compared the proposed method with a BM25 approach, which showed the best accuracy in the related literature. The BM25 is a bag-of-words model similar to tf-idf, which is widely used by search engines to rank a set of documents according to their similarities to a query (Jones et al. 2000). The similarity between a word $w_i$ and another document $d_j$ is calculated as follows.

6 $$\begin{aligned} BM25\; \hbox {Score}(w_i,d_j)= & {} \frac{ f_{ij} \cdot (k_1 + 1) }{ f_{ij} + k_1 \cdot \left( 1 -b + b \cdot \frac{F_j}{F_{\mathrm{ave}}} \right) } \log \frac{|D| - |D_i| + 0.5}{|D_i| + 0.5} \end{aligned}$$

$f_{ij}$ is the frequency of the word $w_i$ in the document $d_j$. $F_j$ is the length of the document $d_j$ in words, and $F_{avg}$ is the average document length in the entire document set. |*D*| is the total number of documents in the set, and $|D_i|$ is the number of documents containing the word $w_i$. $k_1$ and *b* are constants and were set to 2.0 and 0.75, respectively. Note that the latter part corresponds to the inverse document frequency (idf) of the word $w_i$. To compare it with our paragraph vectors, we constructed BM25 vectors in the basic form by aligning BM25 scores of sorted words, instead of summarizing them to get a similarity value. The words were limited to terms included in the JST thesaurus, but if the frequency of a word in the document set is zero, the dimension of the word is reduced. The resulted vectors had 3722 dimensions.

Consequently, Table 1 presents precisions, recalls, and F1-scores for *weak, middle*, and *strong* in both vectors, respectively. Some examples of project pairs and their cosine values are shown in Table 2. We confirmed that the paragraph vectors with entropy clustering obtained an F1-score of 79% in total, which means that 79 project similarities (i.e., distances in the map) matched the majority decisions of the members’ opinions. An F1-score of the BM25 vectors in total remained 20%. The members’ opinions were in “fair” agreement (Fleiss’ Kappa $\kappa$ = 0.29). In more detail, the *strong* pairs that have many sentences and words similar to each other can be easily estimated. However, the weaker pairs had difficulty, since they need to be distinguished from *not-related* pairs and stronger pairs. Examples misjudged include, e.g., the not-related pairs of two projects that have the same acronyms with different meanings, and the stronger pairs of two projects that have only a few common words, but which are recent technologies attracting attention. We expect that those words will eventually have higher entropies and then the project similarities will be estimated to be stronger. We also plan to replace acronyms in project descriptions with full words before making vectors. By contrast, an F1-score of the baseline method in total remained at 21%, since, as described in “Comparison with the baseline on the* unclustered* problem” section, most projects had weaker similarities to fewer projects than those in the proposed method.

**Table 1:** Accuracy of project similarities (%)

Method	PV with entropy			BM25 vector		
Strength	Weak	Middle	Strong	Weak	Middle	Strong
Precision	77.5	83.3	100.0	65.0	60.0	100.0
Recall	98.6	33.3	83.3	18.6	20.0	66.7
F1-score	86.8	47.6	90.9	28.9	30.0	80.0

**Table 2:** Examples of two projects relationship

Title/(desc.)	cos.	Title/(desc.)
Understanding the physics of galaxy formation and evolution at high redshift/understanding the processes regulating galaxy ...	0.50 (weak)	The birth of the first stars and galaxies/the aim of this proposal is to simulate the formation and evolution of galaxies within the ...
Asymptotic graph properties/many parts of graph theory have witnessed a huge growth over the last years, partly because of their relation to theoretical computer science and statistical physics ...	0.52 (weak)	Benj amini-schramm approximation of groups and graphings/large graphs have become central objects in many fields in the last couple of decades: in neural sciences, network sciences ...
A high intensity neutrino oscillation facility in Europe/the recent discovery that the neutrino changes type (or flavour) as it travels through space, a phenomenon referred to as neutrino oscillations,...	0.53 (weak)	Probing fundamental properties of the neutrino at the sno+ experiment/i propose a comprehensive programme of research on sno+, a multi-purpose neutrino experiment that has the capacity ...
Systems biology of pseudomonas aeruginosa in biofilms/systems biology is a new and rapidly growing discipline . it is widely ...	0.54 (weak)	Cyclic-di-gmp: new concepts in second messenger signaling and bacterial biofilm formation/biofilms represent a multicellular ...
Investigation of human nucleoporins stoichiometry and intracellular distribution by quantitative mass spectrometry/the nuclear pore complex (npc) is one of the most intricate multi-protein ...	0.56 (weak)	Atlas of cell-type specific nuclear pore complex structures/the nuclear pore complex (npc) is one of the most intricate components of eukaryotic cells and is assembled from ~ 30 nucleoporins ...
European science and technology in action building links with industry, schools and home/the aim of establish is to facilitate and implement an inquiry based approach in the teaching and learning ...	0.67 (middle)	Science teacher education advanced methods/helping teachers raise the quality of science teaching and its educational environment has the potential to increase student engagement,...
Support to tenth european conference on turbomachinery-fluid dynamics and thermodynamics, lappeenranta, finland, 15–19 march 2013/the european turbomachinery conference is ...	0.99 (strong)	Support to ninth european conference on turbomachinery-fluid dynamics and thermodynamics, istanbul, turkey, 21–25 march 2011/the european turbomachinery conference is ...

**Fig. 7:**
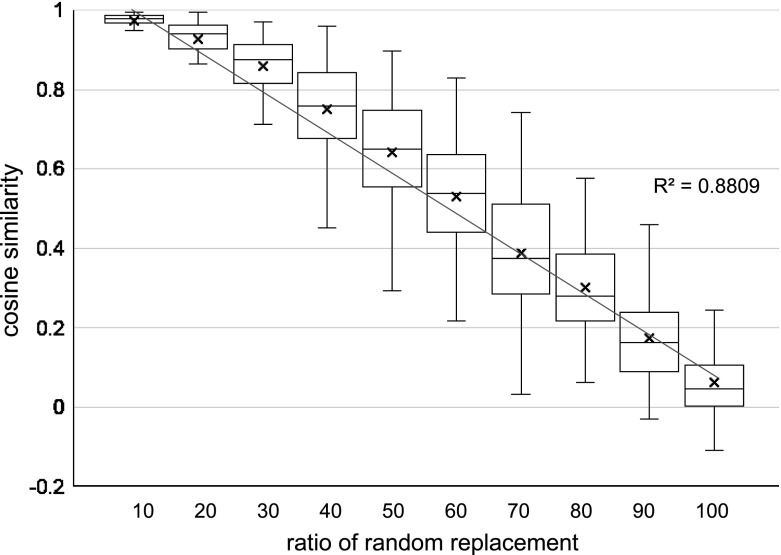
Similarities of artificial data with partial replacement in PV with entropy

**Fig. 8:**
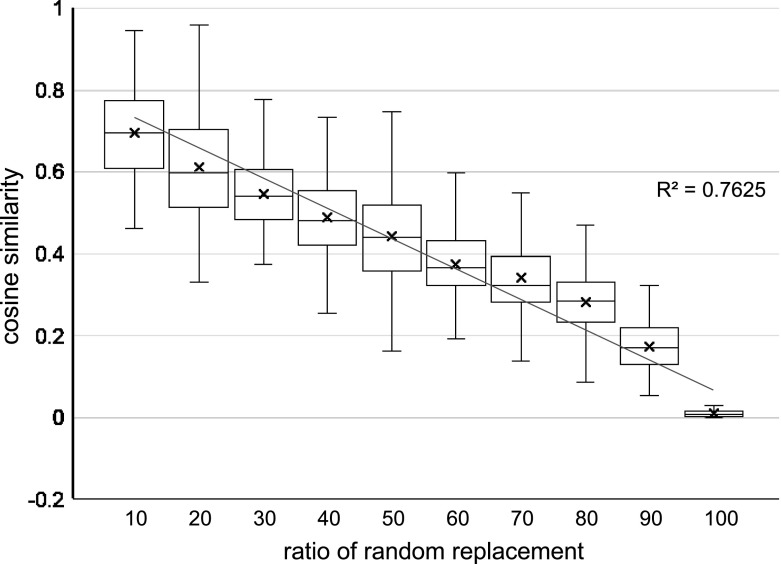
Similarities of artificial data with partial replacement in BM25 vector

Next, we evaluated the accuracy of content similarities using the artificial data, part of which are randomly replaced with the other projects. We replaced 10, 20, ..., 100% of a project description in the FP7 and NSF data set with sentences randomly selected from the other project description. Then, we measured a cosine similarity between a vector generated from the artificial project description and a vector of the original project description. The projects were randomly selected from all projects, and then we evaluated 1,000 pairs of the original project and the artificial project. The relationship of the replacement ratios and the cosine similarities is shown in Fig. 7 and 8.

Consequently, we confirmed that there was an obvious correlation between content similarities of projects descriptions and their cosine similarities with $R^2 = 0.88$. By contrast, the BM25 vectors had a slightly lower value $R^2 = 0.76$, but the problems were that the BM25 vectors could not use the whole range [0...1] of similarity; thus, the resolution of the similarity became lower than the proposed vectors. Moreover, the variances, especially in the lower replacement ratios were larger than the proposed ones. The standard deviation on average from 10 to 50% replacement was $\sigma = 0.08$ in the proposed ones but $\sigma = 0.12$ in the BM25 vectors. We should also note that it is natural that the variance around 50% replacement was greater than those near 10 and 90% replacement, since there are both cases that the replaced sentences are similar to the original sentences by chance, and totally different in them. The paragraph vectors without the entropy clustering in the baseline also had the same trend, but the vectors with the entropy clustering had higher similarities on average. This result is consistent with Fig. 5 and 6 showing the relationships between the cosine similarities and the number of edges.

### Comparison with SIC codes

Finally, we compared our FP7 map with the classification by SIC codes. There is the criticism that discipline-level categories are inaccurate (Klavans and Boyack 2017b); however, since the SIC codes were officially assigned to the FP7 projects, we compared our map with the SIC codes to confirm that the map mainly follows the established classification. In 25,607 FP7 projects, there were little projects without sufficient descriptions. There were 12 projects with less than 100 characters, 31 projects with less than 300 characters, and 110 projects with less than 500 characters. The number of characters of the average was 1802. We made clusters from entropy-based paragraph vectors for the FP7 projects using the simple *k*-means clustering method. The *k* was set to 66 since the number of valid SIC codes in all FP7 project descriptions was 66. Part of the SIC codes of projects that compose each cluster Cl 1...Cl 10 are listed in Table 3. 17

Then, to confirm the statistically significant difference of clusters, we conducted a Chi-squared test on the whole table. As a result, the *p* value was 0.0 (< 2e−16), and the effect size *Cramer’s* *V* was 0.131 (small to medium). Since interdisciplinary projects have multiple SIC codes and also there are similar projects with different codes, each cluster includes several different codes, but we found that each cluster is unique and there is the significant difference between the clusters.

**Table 3:** SIC codes by cluster when splitted into the same number as SIC code (*COO* coordination, cooperation, *EMP* employment issues, *MED* medicine, health, *ENV* environmental protection, *IND* industrial manufacture, *ECO* economic aspects)

SIC	Cl 1	Cl 2	Cl 3	Cl 4	Cl 5	Cl 6	Cl 7	Cl 8	Cl 9	Cl 10
BIO	**156**	20	10	3	109	22	19	2	4	0
SOC	13	**300**	32	15	18	28	35	3	24	48
COO	30	50	**120**	29	26	17	52	10	19	30
EMP	26	95	3	**60**	35	10	51	13	5	11
MED	31	16	18	5	**151**	17	9	3	7	2
INF	2	27	25	36	10	**119**	10	1	92	20
ENV	0	15	35	1	1	14	**148**	3	25	26
IND	3	15	2	39	7	26	10	**51**	43	1
ICT	0	7	14	19	4	77	4	0	**94**	0
ECO	1	19	14	0	4	3	37	8	17	**51**

Bold number indicates the dominant code in each cluster

**Fig. 9:**
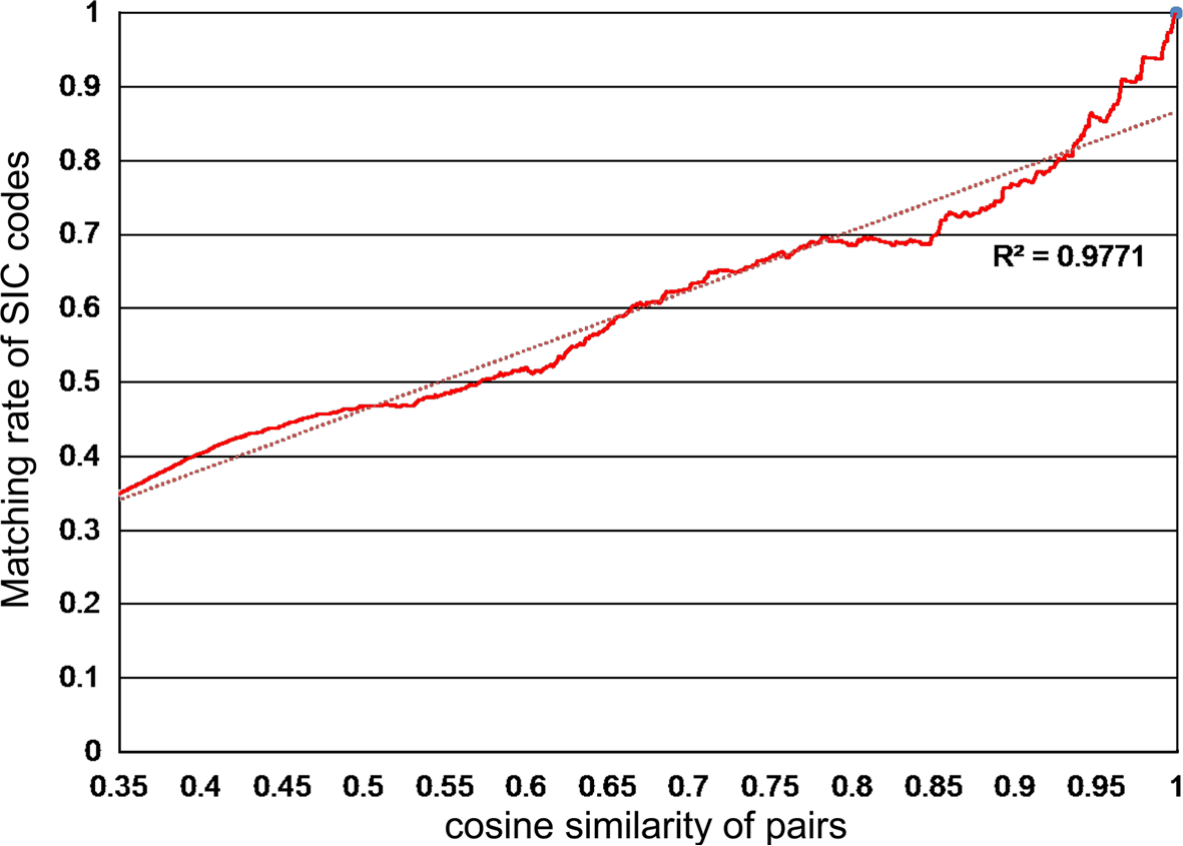
Matching rate of SIC codes in project pairs according to cosine similarities

Moreover, Fig. 9 shows the matching rate of SIC codes between projects pairs according to cosine similarities. Since a project had several SIC codes, we decided that the SIC codes of both projects are matched if one of the codes is matched to one of another project’ codes. In this graph, there is an obvious correlation between cosine similarities of paragraph vectors and SIC code classification.

**Fig. 10:**
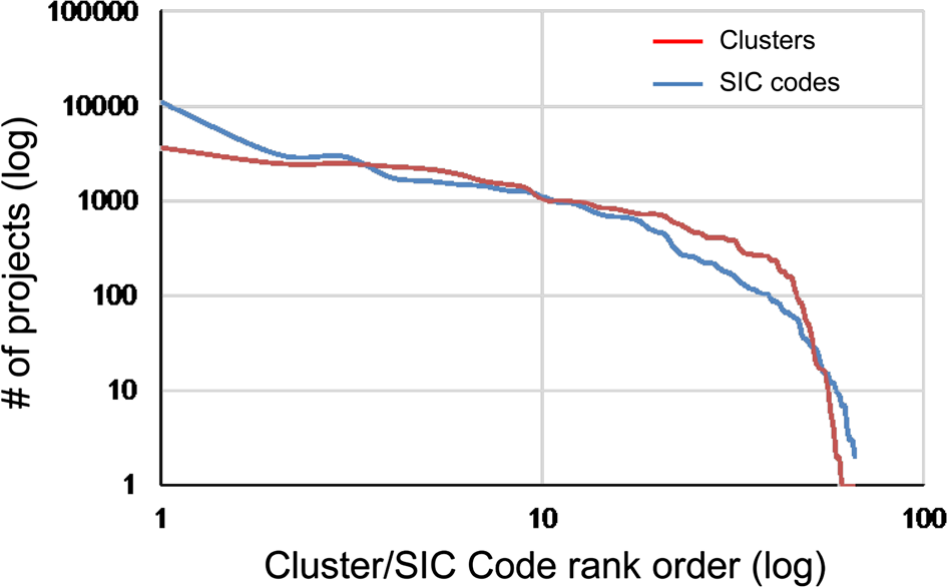
Project distributions by SIC code and by cluster when splitted into the same number as SIC code

Furthermore, Fig. 10 presents the project distributions by the SIC code and by the cluster. In this graph, we can find that the distribution by the SIC code and one by the cluster have the similar trend. Consequently, we observed that our content-based map mainly follows the already-established SIC classification in FP7, although the map help understand the relationships between projects in the same code and in the different codes, e.g., how similar the new interdisciplinary project is to which existing project.

## Conclusion and future work

Navigating the scientific landscape is expected from S&T enterprises, research laboratories, and policy analysts and funding agencies. However, techniques based on citation linkages are not applicable for characterizing the most up-to-date scientific landscape, since project descriptions do not have references and also recently published articles do not have enough citations yet. Therefore, we assessed the relationships using a content-based method, instead of citation analysis. After improving the existing paragraph embedding technique with an entropy-based clustering method, we successfully formed a lattice-like map, which is linked by strings of aligned projects whose subjects are relevant to each other, and we confirmed the good face validity through comparison with the original paragraph vectors, BM25 vectors, and conventional classification codes.

As the next step, we will extract new insights from the map of research projects, especially in comparison with conventional science maps based on citation analysis of published papers. In the future, we could use paragraph vectors for several statistical applications, rather than drawing cosine similarity networks. The result will raise the discoverability of research in the era of open access. We also plan to perform additional comparisons with other project data sets, such as FP7 and JST funding projects. Since concept vectors can be shared across different languages, English and Japanese documents can be compared through the JST thesaurus with English and Japanese notations.

## Footnotes

1 http://cordis.europa.eu/guidance_old/sic-codes_en.html.

2 https://www.acm.org/publications/class-2012.

3 The recently published articles have references but are not cited enough from other articles.

4 https://www.starmetrics.nih.gov/.

5 http://nihmaps.org/index.php.

6 http://cordis.europa.eu/fp7/.

7 https://www.nsf.gov/funding/index.jsp.

8 https://deeplearning4j.org.

9 Due to the page limitation, only the maps of FP7 are shown in this paper.

10 http://jglobal.jst.go.jp/en/.

11 http://thesaurus-map.jst.go.jp/jisho/fullIF/index.html (in Japanese).

12 http://togodb.jst.go.jp/sample-apps/map_FP7/ (Click “Drawing Map” to draw the map. A list of SICs is shown in the lower part of the page.).

13 http://togodb.jst.go.jp/mappingscience/.

14 https://github.com/gephi/gephi/wiki/OpenOrd.

15 Note that the numbers of projects appearing in Figs. 1 and 4 are different because the figures have the same edge threshold.

16 The relationship of < 0.5 had difficulty in determining the relevance for human workers.

17 The first rank of the SIC codes, SCI (Scientific Research) is used for 10,963 of 25,607 projects. We thus omitted the code from the table.
